# Increased transgenerational epigenetic variation, but not predictable epigenetic variants, after environmental exposure in two apomictic dandelion lineages

**DOI:** 10.1002/ece3.3871

**Published:** 2018-02-19

**Authors:** Veronica Preite, Carla Oplaat, Arjen Biere, Jan Kirschner, Wim H. van der Putten, Koen J. F. Verhoeven

**Affiliations:** ^1^ Department of Terrestrial Ecology Netherlands Institute of Ecology (NIOO‐KNAW) Wageningen The Netherlands; ^2^ Laboratory of Nematology Wageningen University Wageningen The Netherlands; ^3^ Institute of Botany of the Czech Academy of Sciences Průhonice Czech Republic

**Keywords:** DNA methylation, drought, Europe, salicylic acid, stress memory, *Taraxacum officinale*

## Abstract

DNA methylation is one of the mechanisms underlying epigenetic modifications. DNA methylations can be environmentally induced and such induced modifications can at times be transmitted to successive generations. However, it remains speculative how common such environmentally induced transgenerational DNA methylation changes are and if they persist for more than one offspring generation. We exposed multiple accessions of two different apomictic dandelion lineages of the *Taraxacum officinale* group (*Taraxacum alatum* and *T. hemicyclum*) to drought and salicylic acid (SA) treatment. Using methylation‐sensitive amplified fragment length polymorphism markers (MS‐AFLPs) we screened anonymous methylation changes at CCGG restriction sites throughout the genome after stress treatments and assessed the heritability of induced changes for two subsequent unexposed offspring generations. Irrespective of the initial stress treatment, a clear buildup of heritable DNA methylation variation was observed across three generations, indicating a considerable background rate of heritable epimutations. Less evidence was detected for environmental effects. Drought stress showed some evidence for accession‐specific methylation changes, but only in the exposed generation and not in their offspring. By contrast, SA treatment caused an increased rate of methylation change in offspring of treated plants. These changes were seemingly undirected resulting in increased transgenerational epigenetic variation between offspring individuals, but not in predictable epigenetic variants. While the functional consequences of these MS‐AFLP‐detected DNA methylation changes remain to be demonstrated, our study shows that (1) stress‐induced transgenerational DNA methylation modification in dandelions is genotype and context‐specific; and (2) inherited environmental DNA methylation effects are mostly undirected and not targeted to specific loci.

## 1 INTRODUCTION

Epigenetic modifications, such as DNA methylation, can affect gene activity without changing the underlying DNA sequence and are involved in transposable elements (TEs) silencing (Lippman & Martienssen, 2004). Exposure to biotic and abiotic stress has been shown to alter DNA methylations (Aina et al., 2004; Choi & Sano, 2007; Cramer, Urano, Delrot, Pezzotti, & Shinozaki, 2011), and some of the induced DNA methylation modifications are transmitted to successive generations where they might mediate phenotypic effects (Bilichack et al., 2015; Boyko et al., 2007; Cheng, Hockman, Crawford, Anderson, & Shiao, 2004; Kou et al., 2011; Verhoeven, Jansen, van Dijk, & Biere, 2010; Wibowo et al., 2016). Such a transgenerational “memory” of stress has been proposed to play a role in adaptation by generating epigenetic variants that are specifically tolerant to the environmental stress that triggered them (Lämke & Bäurle, 2017; Luna, Bruce, Roberts, Flors, & Ton, 2012; Rasmann et al., 2012). However, support for this hypothesized adaptive role of DNA methylation is very limited and requires further empirical studies (Pecinka & Scheid, 2012).

To be transgenerationally effective, epigenetic information needs to be transmitted through genome resetting and reprograming during gametogenesis and zygote development. Unlike in mammals, in plants, a considerable part of the DNA methylations is meiotically stable (Feng, Jacobsen, & Reik, 2010) or may be transmitted between generations via small RNAs that could guide re‐establishment of parental DNA methylation patterns in offspring (reviewed in Bond & Baulcombe, 2014 and Iwasaki & Paszkowski, 2014). Indeed, sRNAs were found to be required to sustain induced defense responses against herbivory across generations in Arabidopsis using a sRNA biogenesis mutant (Rasmann et al., 2012). Although recent studies are providing first estimates of the rate and transgenerational stability of spontaneous DNA methylation modifications (Becker et al., 2011; Van der Graaf et al., 2015), it remains unclear to what extent the rate of heritable modifications is affected by stress exposure, and for how many generations DNA methylations can persist. It is also unclear what level of persistence is necessary to have an important impact on adaptive processes (Herman, Spencer, Donohue, & Sultan, 2013; Herman & Sultan, 2011; Rapp & Wendel, 2005).

DNA methylation variants can arise spontaneously, as a consequence of environmental inputs, or can be under nearby (cis) or distant (trans) genetic control. In natural *Arabidopsis* accessions, a large proportion of natural DNA methylation variants are under such genetic control (Dubin et al., 2015). However, a portion of methylation variants can also be autonomous, independent of genetic variation (“pure” epigenetic variants, sensu Richards, 2006), and thus potentially relevant for adaptation in ways that cannot be explained by sequence variation alone (Bossdorf, Richards, & Pigliucci, 2008; Richards, 2006). In practice, it is difficult to distinguish autonomous from genetically‐mediated epigenetic variation as it is possible that genetic changes that influence a particular epigenotype remain undetected (Johannes et al., 2009; Richards, 2006, 2011). Populations that lack significant genetic variation, such as asexually propagating lineages, might therefore be well suited to investigate the potential of autonomous epigenetic inheritance (Bossdorf et al., 2008). One can speculate that such epigenetic variation contributes to the ecological success of some asexual invaders that colonize vast areas as a single dominant genotype (Ahmad, Liow, Spencer, & Jasieniuk, 2008; Hollingsworth & Bailey, 2000; Zhang, Zhang, & Barrett, 2010).

To investigate heritable DNA methylations, we used apomictic, that is asexually reproducing, dandelions of *Taraxacum* Wigg. sect. *Taraxacum* (commonly called *Taraxacum officinale* Wigg., see Kirschner & Štěpánek, 2011). Dandelions show geographic parthenogenesis where the distribution of apomictic lineages extends beyond the distribution limits of sexually reproducing dandelions toward northern regions. In Europe, many different obligate apomictic lineages colonized northern regions after the retreat of land ice, approx. 10,000 years ago (Comes & Kadereit, 1998). This particular geographical distribution pattern provides a natural study system of widespread apomictic dandelion lineages, with each lineage harboring limited potential to adapt through genetic variation. Previous research on a newly synthesized apomictic dandelion genotype showed that stress exposure can cause DNA methylation changes and moreover, that these changes could be stably transmitted to the next generation (Verhoeven, Jansen, et al., 2010). This study aimed to investigate the persistence and the generality of inheritance of stress‐induced epigenetic modification in apomictic dandelion lineages.

To study stress‐induced heritable DNA methylations, we carried out a controlled experiment exposing apomictic dandelions to two different stresses and investigated the persistence of induced methylation changes in two subsequent unexposed generations. Two apomictic dandelion lineages were used that were collected from three different sites which we hereafter abbreviate as FI (Finland, high‐latitude site), CZH (East Czech Republic, the Carphathians, medium‐altitude site), and CZL (Central Czech Republic, the Bohemian lowlands, low‐altitude site). As northern and mountainous regions may represent more stressful environmental conditions, we hypothesized that at the FI and the CZH site, plants may have been selected for higher levels of plasticity that might be partly mediated by a higher capacity for stress‐induced methylation modifications.

As for abiotic stress, we used drought and salicylic acid (SA), which is a plant hormone involved in several processes including defense signaling in response to pathogens (Delaney et al., 1994; Vicente & Plasencia, 2011). Drought and SA‐induced stress represent important environmental factors for plants in all sampling regions in Central Bohemia, the White Carpathian region, and South Finland. Spring droughts occur regularly, although in relatively mild form, in Czech Republic and in continental Finland (Potop, Boroneanţ, Možný, Štěpánek, & Skalák, 2014). Pathogen pressure is a very common biotic stress and intensifies toward lower latitudes in Europe (Schemske, Mittelbach, Cornell, Sobel, & Roy, 2009; Verhoeven & Biere, 2013). Moreover, these stresses are predicted to become more severe and frequent as the current climate change proceeds (IPCC 2013; Pautasso, Dӧring, Garbelotto, Pellis, & Jeger, 2012).

Based on methylation‐sensitive amplification polymorphisms (MS‐AFLPs) that detect DNA methylation variation at genomewide anonymous marker loci, we specifically tested three hypotheses: (1) upon stress application DNA methylation patterns change, (2) these methylation modifications are inherited to next generations, and (3) plant accessions that originate from higher latitude and altitude sites show a higher capacity for stress‐induced methylation modifications.

## 2 MATERIAL AND METHODS

### 2.1 Study species

The apomictic common dandelion of sect. *Taraxacum*, the *T. officinale* group, is a widespread perennial forb in lawns, meadows, and pastures that has spread worldwide, especially in temperate zones but also reaching into subpolar and alpine zones (Richards, 1973). Dandelions form taproots with rosettes and produce wind‐dispersed seeds. In apomictic dandelions, these seeds are produced from unreduced egg cells via embryogenesis without fertilization by male gametes (diplospory, parthenogenesis). Likewise, the endosperm develops autonomously without fertilization (Koltunow, 1993). Generally, apomicts are polyploid (Asker & Jerling, 1992; Mogie & Ford, 1988). In the case of *T. officinale*, the apomicts are mostly triploid while the sexuals are diploid (Richards, 1973, 1989; Riddle & Richards, 2002). New apomictic lineages arise in mixed populations of apomictic and sexual dandelions when pollen from apomicts fertilizes sexual dandelions (Richards, 1973), resulting in offspring of various ploidy levels, some of which are functionally apomicts (Tas & Van Dijk, 1999). In the regions without sexual common dandelions, local populations consist of few to numerous distinct apomictic lineages, morphologically and genetically recognizable entities, sometimes referred to as microspecies, under binomials. Hundreds of microspecies within the *T.officinale* group have been described in Europe (Kirschner & Štěpánek, 2011).These apomictic dandelion lineages are often widespread with a distribution that extends from western to eastern Europe, and from the southern Central Europe to Northern Europe. The distribution pattern in the sect. *Taraxacum* resembles a classical geographic parthenogenesis, as the distribution of the apomicts extends beyond that of the sexually reproducing dandelions (Menken, Smit, Nijs, & Den Nijs, 1995; Verduijn, Van Dijk, & Van Damme, 2004).

### 2.2 Plant material and growing conditions

**Figure 1:**
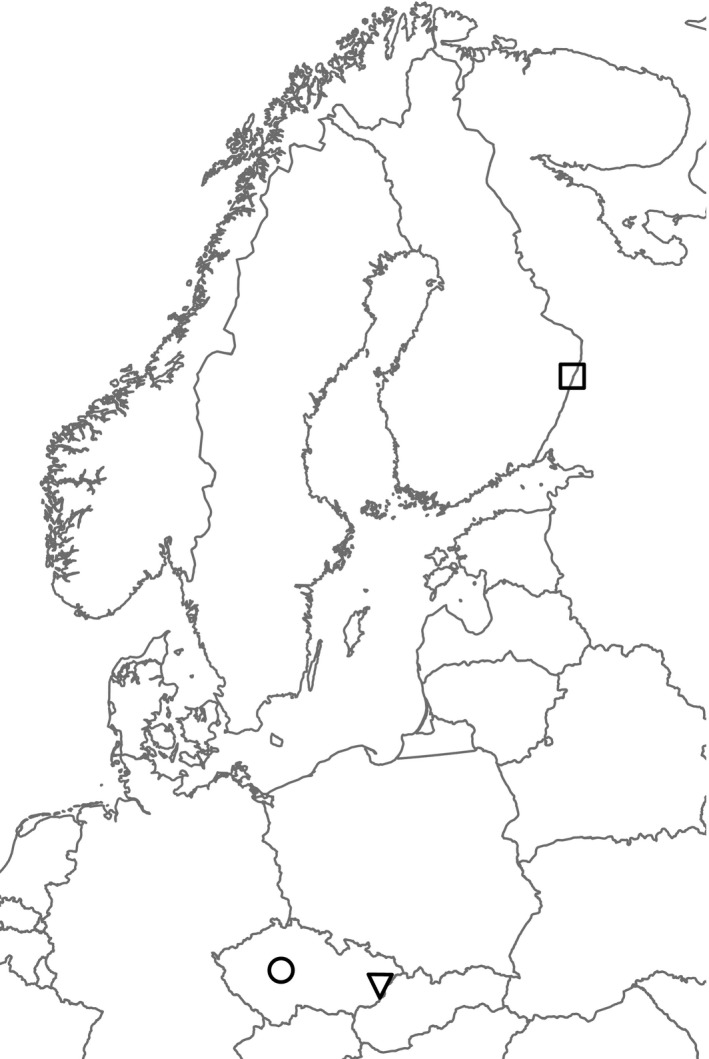
Map of the sampling sites. Seeds of *Taraxacum alatum* and *Taraxacum hemicyclum* were collected in the Bohemian lowlands (CZL, circle), the Carpathians (CZH, triangle) and in Finland (FI, rectangle)

Seeds were collected from two widespread apomictic dandelion lineages: *T. alatum* H. Lindb. and *T. hemicyclum* G. E. Haglund. Seed heads were collected in spring 2013 from three locations in North‐Eastern Europe: from two locations in Czech Republic which differed in elevation and from one location in Finland (Figure 1). Throughout this study, we refer to the descendants of a single field‐sampled individual as an accession. The collection of seeds in the field was carried out by taxonomic specialists that recognize these geographically widespread *Taraxacum* microspecies by specific phenotypic traits. The consistent ability to identify the apomictic clone clusters as individual microspecies by means of their phenotypes was proven in Kirschner et al. (2016). After having the seeds propagated for one generation under common greenhouse conditions, we confirmed the clonal identity of the *T. alatum* and *T. hemicyclum* plants with eight microsatellite markers which showed nearly identical multilocus genotypes for all accessions within a microspecies (Table S2).

Throughout all generations of the experiment, we used the same protocol for seed collection and seed sterilization and the same temperature and light conditions for the germination, growth, and vernalization stages. Seeds derived from the first produced seed head per plant; seeds were surface‐sterilized for 5 min with 0.5% sodium hypochlorite including 0.05% Tween20 (Sigma‐Aldrich, Zwijndrecht, the Netherlands) and afterward washed with demineralized water. Sterilized seeds were germinated on 0.8% agar plates for 10 days (14 hr light/10 hr dark, 18°C/14°C, 60% relative humidity on average, daylight maintained at a minimum of 30 μmol/m^2^/s). Seedlings were individually transplanted to 9 × 9 × 10 cm pots containing a mixture of 80% potting soil and 20% pumice that was equalized to 210 ± 5 g. Nutrients were supplied with 1.5 g of Osmocote granules (15 N + 3.5 P + 9.1 K + 1.2 Mg + trace elements; Osmocote exact Mini, Everris international BV, the Netherlands). Afterward, the seedlings were grown under the same condition as during germination but with a light level of approximately 315 μmol/m^2^/s and were watered several times per week, depending on the rate of water loss. Prior to vernalization, rosette leaves were clipped back to 4–5 cm and the plants were put in a cold room at 4°C (16 hr daylight) for 5 weeks, with occasional watering depending on moisture loss.

### 2.3 Stress experiment

For each of the six accessions used in this study (2 apomictic lineages × 3 sampling sites), seeds were derived from a single greenhouse‐propagated individual. Thirty‐six seedlings per accession were distributed over control, drought stress and salicylic acid (SA) stress (12 replicate plants per treatment). All plants of *T. alatum* were grown together in one climate chamber, and all plants of *T. hemicylcum* were grown in another climate chamber with identical settings. In each growth chamber, plants from all three accessions within a treatment group (control, drought, salicylic acid) were randomized within treatments. Plants from a treatment group (control, drought, salicylic acid) were placed in rows to ensure nontouching between the treatment groups. After 4 weeks of growth in the climate chamber, the drought stress started: water was withheld from the “drought” treatment until at least 80% of all “drought” plants showed wilted leaves, at which moment, all “drought” pots were fully saturated with water. While the other groups were regularly watered, the “drought” group experienced this deprivation of water ten times within a period of 4 weeks. After 5 weeks of growth, a one‐time SA treatment was applied: 0.5 ml of a 10 mmol/L SA solution (Sigma S‐7401, dissolved in 0.1% Triton X‐100 surfactant solution, pH = 2.3) was spread over three medium‐sized leaves. The third, control, group received no treatment, also no mock treatment, as these plants were also used as control for the drought treatment. The absence of a mock treatment implies that we cannot control for potential artifacts arising from the surfactant solution. After 8 weeks of growth, leaf punches were collected from the third fully developed leaf of each individual plant and put on ice for subsequent DNA isolation. Subsequently, the plants were moved to a cold room for vernalization. All plants flowered approximately 6 weeks after the end of the vernalization period and seeds were collected from each plant. Using single‐seed descent, the subsequent two generations, G2 and G3, were grown under common control conditions in the greenhouse following the same experimental design and separated per genotype as described for G1. For the drought experiment, we evaluated DNA methylation for all plants in G1 and G3, to specifically address the question whether drought‐induced DNA methylation changes exist that persist for two subsequent unexposed generations. For the SA experiment, we evaluated DNA methylation in all three generations, but we limited this analysis to only one accession, the northern accession (FI). DNA was isolated from leaf punches taken after 7 weeks of growth for G2 and taken after 4 weeks of growth in G3. G3 plants were sampled at an earlier stage than G2 plants because this is optimal for high‐quality DNA extraction. G2 plants were required to grow to a larger size before sampling in order to ensure unaffected post‐sampling growth and seed set. It was previously shown that dandelion leaf tissue MS‐AFLP profiles are highly similar between plants from different ages (Verhoeven, Van Dijk, & Biere, 2010).

### 2.4 DNA isolation and MS‐AFLP

DNA was isolated following the CTAB procedure by Rogstad (1992) with minor modifications (Vijverberg, Van der Hulst, Lindhout, & Van Dijk, 2004) using approximately 1 cm^2^ of fresh leaf tissue. During sampling, the leaf tissue was kept on ice in microtubes containing two 1/8″ steel balls and after grinding, the samples were homogenized in CTAB buffer using a Tissuelyser II (Qiagen, the Netherlands) followed by washing and DNA precipitation steps. The final DNA pellet was dissolved in 50 μl TE and stored at −20°C until DNA was collected for all generations.

For the MS‐AFLP analysis, the isolated DNA was digested with the methylation‐sensitive enzymes *Hpa*II as frequent cutter and *Eco*RI as rare cutter following Keyte, Percifield, Liu, and Wendel (2006) with some modifications. *Hpa*II recognizes the tetranucleotide sequence, 5′‐CCGG, which can be methylated on one or both DNA strands and at the internal and/or external cytosine. *Hpa*II cuts if the restriction site is free from methylations or if the external cytosine is hemi‐methylated (e.g., see Schulz, Eckstein, & Durka, 2013). Usually MS‐AFLPs are run with a combination of the methylation‐sensitive restriction enzymes *Hpa*II and *Msp*I, which enables the distinction between methylation polymorphisms and DNA sequence polymorphisms. However, in samples where genetic variation can be assumed to be negligible, such as under apomictic reproduction as in our experiment, variation in *Hpa*II and *Msp*I fingerprint profiles can be interpreted directly as methylation polymorphisms (Verhoeven, Jansen, et al., 2010). We therefore used only *Hpa*II to capture methylation variation. Based on previous testing, we selected eight *Eco*RI/*Hpa*II primer combinations (Table S3). The digestion mix contained ten units of each *Eco*RI (100,000 U/ml) and *Hpa*II (50,000 U/ml) and the corresponding buffer (all from New England BioLabs, 180 Bioke, the Netherlands) in a total volume of 20 μl containing 50 ng of DNA. The digestion ran for three hours at 37°C. Afterward, adapters were ligated in a total reaction volume of 30 μl containing: 1 Unit of T4 DNA ligase and ligase buffer (ThermoFisher scientific, the Netherlands), 3.75 pmol of *Eco*RI adapter, and 37.5 pmol of *Hpa*II adapter for 18 hr at 22°C followed by 10 min at 65°C. The ligation product was diluted to 15% in water (Sigma‐Aldrich, the Netherlands). Preamplification was performed in a total volume of 50 μl using: 1× buffer, 125 nmol MgCl_2_, 2.5 U Taq DNA polymerase (all from GC biotech BV, the Netherlands), 10 nmol dNTPs (ThemoFisher scientific), 15 pmol of each pre‐selective primer, and 10 μl of diluted ligation product. The reaction started with 2 min hold at 72°C followed by 20 cycles of 30 s at 94°C, 30 s at 56°C, 2 min at 72°C and finished with 10 min incubation at 60°C and hold at 10°C. These pre‐amplified products were diluted to 5% and proceeded to the selective amplifications in a total volume of 25 μl containing: 1× buffer, 37.5 nmol MgCl_2_, 1.25 U Taq DNA polymerase (all from GC biotech B.V., the Netherlands), 7.5 nmol dNTPs (ThermoFisher scientific, the Netherlands), 10 μg BSA, 5 pmol labeled selective *Eco*RI primer, 20 pmol selective *Hpa*II primer, and 5 μl diluted pre‐amplified product. The selective amplification was started with 2 min hold at 94°C, followed by 10 cycles of 30 s at 94°C, 30 s at 65°C, 2 min at 72°C and 25 cycles with 30 s at 94°C, 30 s at 56°C, 2 min at 72°C and ended with 10 min at 60°C before hold at 10°C. The final PCR product was diluted to 2.5% in sterile water and analyzed on an ABI 3130 genetic analyzer (Life Technologies Europe BV, the Netherlands).

MS‐AFLPs were screened in a total number of 320 plants (10 replicate plants per treatment, accession and generation), of which, 317 plants yielded readable MS‐AFLP fragments. Within each apomictic lineage, all selected samples were run through the MS‐AFLP laboratory protocol in fully randomized order. We used for all samples of an apomictic lineage one digestion mix and after digestion proceeded directly with the ligation and pre‐amplification steps. Technical duplicates of MS‐AFLP analysis were performed for a randomly chosen subset of 15% samples in order to quantify the MS‐AFLP error rates, and negative controls were included (10%) to check for peaks that indicate contamination signals and carry‐over effects (Bonin, Ehrich, & Manel, 2007).

### 2.5 Fragment scoring

Fragments between 100 and 500 base pairs were scored using GeneMapper 5.0 (Life technologies Europe BV, NL). Using overlaying peak profiles in GeneMapper, polymorphic loci were identified and included if at least one of the samples showed a peak height exceeding 25. After visually checking each locus, and depending on local peak “signal” and “noise” characteristics which differed considerably between loci, a threshold peak height of either 25 or 50 was chosen to score individual peaks as “present” if peak height exceeded the threshold. Loci were discarded if they were monomorphic or if they contained fragments that showed up in any of the negative controls. Following other MS‐AFLP studies (Alonso, Pérez, Bazaga, Medrano, & Herrera, 2016; Cara, Marfil, & Masuelli, 2013; also see Zhang & Hare, 2012), loci were also discarded if they showed too many mismatches among technical duplicates: We allowed a maximum of three mismatches among the set of 24 pairs of technical duplicates. The averaged mismatch error rate (±standard deviation) across all primer combinations used was before purging for *T. alatum* 8.46 ± 1.70% (*N *=* *65) and for *T. hemicyclum* 9.20% ± 1.39% (*N *=* *72). The retained loci for *T. alatum* resulted in error rates of 1.65 ± 0.46% (*N *=* *49) and for *T. hemicyclum* 2.72 ± 0.55% (*N *=* *53).

### 2.6 Statistical analysis

Within apomictic lineage and per generation, the status of each single marker was analyzed using logistic regression models to test for significant stress and accession effects (R‐function glm() with binomial error distribution and logit link function). *p*‐values were corrected for multiple testing using false discovery rate control at FDR = 0.05 (R‐function p.adjust()). Multivariate analyses were performed based on pairwise distances calculated by counting the absolute number of inconsistent loci between individuals (R‐function designdist()). Based on this distance matrix, permutational multivariate analysis of variance (R‐function adonis()) and analysis of multivariate homogeneity of group dispersions were performed (R‐function betadisper()). The former analysis tests for different mean positions of experimental groups in multivariate MS‐AFLP space while the latter analysis tests for differences between experimental groups in their amount of MS‐AFLP variation irrespective of group mean positions. A principal coordinate analysis was plotted to visualize the multidimensional data (R‐function pcoa() from package Ape with the *x*‐axis jittered to show overlapping samples).

To track individual methylation changes over generations, we first inferred a consensus epigenotype (following Verhoeven, Jansen, et al., 2010), which represents the hypothesized MS‐AFLP profile at the beginning of G1 for all plants from the same accession. We defined this consensus as the methylation state that was observed in plants from the control treatment in G1, for each accession separately, including only loci for which none or maximum one of the 10 replicate plants showed a deviating marker status. This criterion excluded 1–3 loci per accession from the consensus analysis because they were too polymorphic across the control G1 group to confidently call the consensus state. Any deviations of the detected MS‐AFLP from the consensus that were observed in stress treatments and later generations were assumed to have arisen during the experiment. These methylation changes were counted and checked for their persistence in the next generations. For each accession separately, we fitted a generalized linear mixed model to test for effects of generation, G1 treatment, and the interaction generation × G1 treatment on the plant's proportion of MS‐AFLP loci that deviated from consensus (PROC GENMOD in SAS 9.2, using type 3 analysis and likelihood ratio tests for significance).

## 3 RESULTS

### 3.1 Drought and accession effects on methylation

**Figure 2:**
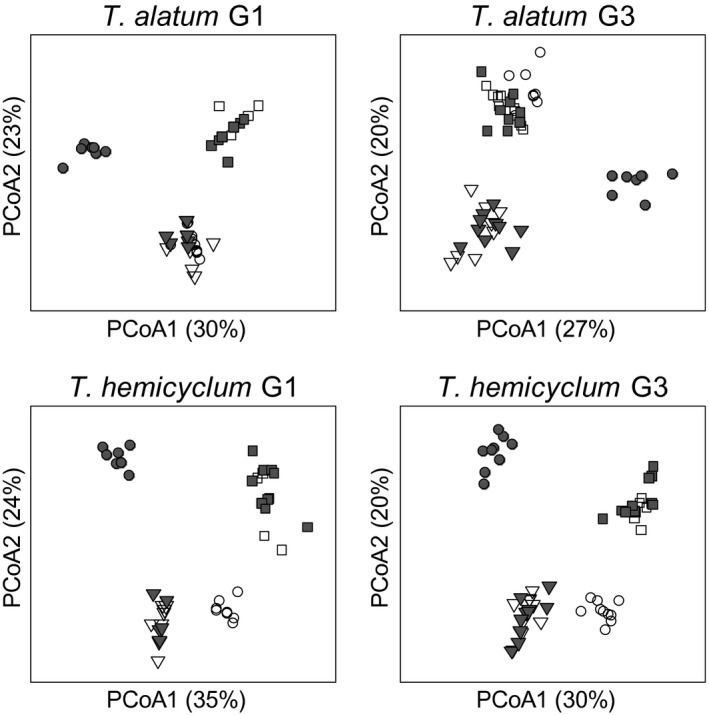
Principal Coordinate Analysis (PCoA) based on MS‐AFLP profiles of drought‐stressed (gray symbols) and control plants (no fill) in the first, stressed generation (G1) and the progeny (G3) that were grown for two generations under unstressed conditions in two apomictic dandelion lineages (*Taraxacum alatum* and *Taraxacum hemicyclum*). The symbols indicate the three accessions: CZL (circle), CZH (triangle) and FI (rectangle)

The DNA methylation patterns (based on *Hpa*II MS‐AFLP profiles) clustered by accession but not by stress treatment: No clear differentiation was found between the methylation profiles of drought‐stressed and control plants (Table 1, visualized in Figure 2). However, in both apomictic lineages, the drought × accession interaction in the first generation was marginally significant (*T. alatum p*‐value = 0.059, *T. hemicyclum p*‐value = .074), suggesting that a weak drought effect may be present but not equally expressed in all accessions. Visual inspection of the PCoA clustering with group centroids in the first generation (Figure S1) indicated that for *T. alatum,* the lowland Czech accession (CZL) may be most responsive to drought while for *T. hemicyclum* the northern (FI) and medium‐altitude (CZH) accession might be more responsive. But even in these accessions, the response was weak, and any accession‐dependency of the response to drought was not inherited, since the interaction effect had disappeared in the third generation.

**Table 1:** Proportion of variance explained (R2) in MS‐AFLP profiles of each generation G1–G3 by accession and stress treatments as determined by permutational multivariate analysis of variance in two apomictic dandelions lineages (*Taraxacum alatum* and *Taraxacum hemicyclum*). Significance is determined based on 10,000 permutation steps (function adonis () from R‐package Vegan)

	*df*	*T. alatum*			*T. hemicyclum*		
		G1	G2	G3	G1	G2	G3
Drought experiment							
Accession	2	0.91***		0.81***	0.91***		0.83***
Drought	1	0.001 ns		0.008 ns	<0.001 ns		0.002 ns
Accession × Drought	2	0.007 **.**		0.003 ns	0.006 **.**		0.005 ns
Salicylic acid experiment							
SA	1	0.073 ns	0.032 ns	<0.001 ns	0.049 ns	0.058 ns	0.045 ns

*p* values significance labels: ***p < 0.001; ns = not significant (*p* ≥ 0.1).

**Figure 3:**
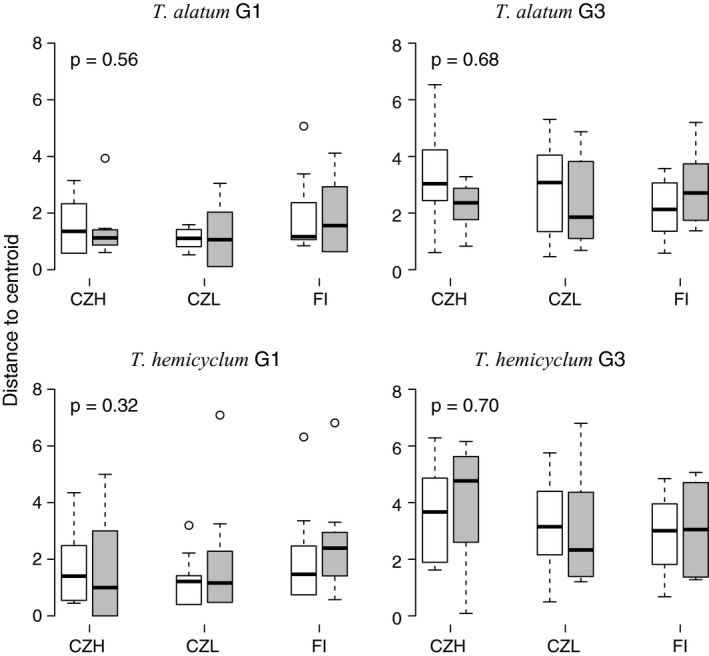
Effects of drought stress and accessions (CZH, CZL, FI) on within‐group variation in MS‐AFLP profiles between replicate plants, calculated as distances to group centroid in multivariate dispersion analysis, in two apomictic dandelion lineages (*Taraxacum alatum* and *Taraxacum hemicyclum*) from the first (G1, stress‐exposed) generation and the third (G3, unexposed) generation. From left to right, the boxplots show the distances to centroid of the three accessions either in white boxplots (control) or gray boxplots (drought stress). *p* Values indicate significance of the treatment effect based on a permutation test with all accessions pooled together

Besides causing a directed shift in methylation variation, treatments might also trigger an increased level of undirected (random) methylation changes. An increase in the number of random changes would promote differentiation in methylation profiles between replicate plants from the same experimental group. However, no such effect was observed in response to drought stress: Multivariate dispersion did not differ significantly between control and drought groups (Figure 3). Nevertheless, despite the lack of an inherited treatment effect, a clear buildup of methylation variation was observed between the first and third generation in both the control and the drought groups (Figure 3). When pooled over treatments in order to test for generation differences, multivariate dispersion analysis revealed a significant increase in DNA methylation variation over generations for four of the six accessions: lowlands (CZL: *T. alatum p*‐value: .003, *T. hemicyclum p*‐value: .006) and medium altitude (CZH: *T. alatum p*‐value: .014, *T. hemicyclum p*‐value: .002); not significant for high latitude (FI: *T. alatum p*‐value: .139, *T. hemicyclum p*‐value: .198).

In addition to these multivariate analyses, we also performed a marker‐by‐marker analysis to test if MS‐AFLP marker status associates with treatment or accession. After controlling for multiple testing at a false discovery threshold of 0.05, the single marker testing revealed that approximately a third of the analyzed loci show an accession effect (*T. alatum*: 16 loci in G1 and 17 loci G3, *T. hemicyclum*: 20 loci in G1 and 19 loci in G3), but none showed a significant drought effect.

### 3.2 Salicylic acid effect on methylation

**Figure 4:**
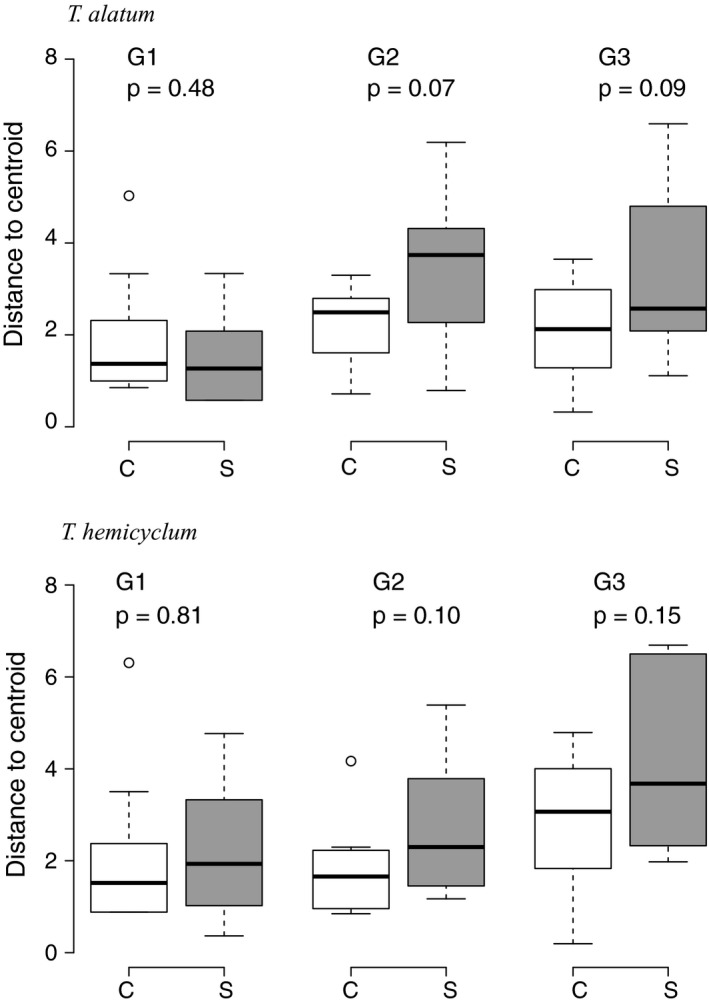
Effects of salicylic acid stress on within‐group variation in MS‐AFLP profiles between replicate plants, calculated as distance to group centroid in multivariate dispersion analysis in two apomictic dandelion lineages (*Taraxacum alatum* and *Taraxacum hemicyclum*, accession FI) from the first (G1, stress‐exposed) and the second and third (G2 and G3, unexposed) generation. From left to right, the boxplots show the distances to centroid separated in white boxplots (control) or gray boxplots (salicylic acid stress). *p* Values indicate significance of the treatment effect based on a permutation test

The multivariate analysis of methylation variation following salicylic acid (SA) application (high‐latitude FI accessions only) showed no overall distinction between the control group and the SA‐stressed plants, neither in the first generation that experienced the stress, nor in the subsequent generations (Table 1). Multivariate dispersion analysis (distance to centroid) showed no significant difference between SA‐stressed and control plants, although a marginally significant trend was observed that offspring of SA‐treated plants showed increased levels of dispersion compared to offspring of control plants (Figure 4). As in the analysis of drought stress (see above), we observed a buildup of DNA methylation variation over generations (pooled across control and stress groups, the generation effect for *T. alatum*:* p*‐value = .017; for *T. hemicyclum*:* p*‐value = .009).

The single marker tests revealed that only a few loci showed a response to salicylic acid treatment (*T. alatum*: three loci in G2 and three loci in G3, *T. hemicyclum*: 1 locus in G3), however, these results did not stand up to the multiple testing correction.

### 3.3 Tracking deviations from consensus

By comparing the status of individual MS‐AFLP markers to an accession‐specific consensus profile, which was based on G1 control plants, individual loci could be identified that showed a methylation change during the experiment. In both the drought and the SA experiments, methylation deviations were observed with a frequency of approximately 1%–3% in G1 and up to 4%–9% in G3 (Tables 2 and 3). For *T. hemicyclum,* the total number of deviations from consensus per individual was significantly higher in the SA‐treated plants and SA‐descendants than in control plants and control‐descendants (*p* < .05; Table 4). This SA effect was also marginally significant in *T. alatum* (*p*‐value: .086; Table 4). No effect of drought stress was detected on the number of methylation changes per individual (Table 5). These analyses, that test deviations from the MS‐AFLP consensus profile established for control plants in G1, were performed across all generations, meaning that the observed SA effect is not necessarily restricted to the first generation. In fact, the frequency of deviations from the consensus profiles showed more pronounced differences between control and SA group in G2 and G3 compared to G1 (Tables 2 and 3). For both drought and SA stress, the generation effect on deviations from the consensus was highly significant (Tables 4 and 5), showing increasing deviations from the consensus from G1 to G3 (see also Figure 2; consistent with a buildup of methylation variation across generations). Of the methylation changes that occurred in G1 in response to drought or SA, 13%‐36% were observed to remain in the changed state until the G3 generation (Tables 2 and 3).

**Table 2:** Number of DNA methylation changes, defined as MS‐AFLP locus presence/absence polymorphisms compared to an accession‐specific consensus epigenotype, in two apomictic dandelion lineages, as affected by drought stress (G1) and after two subsequent generations of propagation in a common environment (G3). In *Taraxacum alatum*, 49 marker loci were evaluated in 160 samples and in *Taraxacum hemicyclum* 53 marker loci were evaluated in 160 samples

	G1	G3	Transmitted to G3^a^
*T. alatum*			
Total cases (markers × samples)	1,353	1,306	
Changes in Control cohort	16	51	4
Changes in Drought cohort	22	47	8
*T. hemicyclum*			
Total cases (markers × samples)	1,570	1,570	
Changes in Control cohort	25	124	2
Changes in Drought cohort	47	130	10

a Deviations from the consensus epigenotype that were observed in G1 and that had not reverted to consensus in G3.

**Table 3:** Number of DNA methylation changes, defined as MS‐AFLP locus presence/absence polymorphisms compared to an accession‐specific consensus epigenotype, in two apomictic dandelion lineages, as affected by salicylic acid stress (G1) the two subsequent generations of propagation in a unstressed common environment (G2 and G3). In *Taraxacum alatum*, 49 marker loci were evaluated in 160 samples and in *Taraxacum hemicyclum* 53 marker loci were evaluated in 160 samples

	G1	G2	Transmitted to G2^a^	G3	Transmitted to G3^a^
*T. alatum*					
Total cases (markers × samples)	460	460		460	
Changes in Control cohort	7	12	**3**	10	**1**
Changes in SA cohort	6	24	**1**	21	**1**
*T. hemicyclum*					
Total cases (markers × samples)	530	530		530	
Changes in Control cohort	12	16	**2**	35	**0**
Changes in SA cohort	16	26	**4**	46	**2**

a Deviations from the consensus epigenotype that were observed in G1 and that had not reverted to consensus in G2 and G3 respectively.

**Table 4:** Generation and salicylic acid (SA) effect on the number of DNA methylation changes per individual, defined as MS‐AFLP (*Hpa*II) locus presence/absence polymorphismus compared to an accession‐specific consensus epigenotype, in two apomictic dandelion lineages (accession FI)

	*df*	*Taraxacum alatum*Chi‐Square	*Taraxacum hemicyclum*Chi‐Square
Generation	2	10.60**	30.28***
SA	1	2.95 **.**	3.95*
Generation × SA	2	2.11 ns	0.31 ns

Chi‐square and significance from generalized linear mixed model tests.

*p* values significance labels: ***p <0.001; **p <0.01; *p <0.05; . *p* <0.1; ns = not significant (*p* ≥0.1).

**Table 5:** Generation and drought effect on the number of DNA methylation changes per individual, defined as MS‐AFLP (*Hpa*II) locus presence/absence polymorphismus compared to an accession‐specific consensus epigenotype, in two apomictic dandelion lineages and three accessions (CZH, CZL, FI)

Accession	*Taraxacum alatum* Chi‐Square	*Taraxacum hemicyclum* Chi‐Square
Generation		
CZH	14.31***	55.62***
CZL	16.33***	44.25***
FI	2.00 ns	21.70***
Drought		
CZH	0.06 ns	2.64 ns
CZL	0.17 ns	1.81 ns
FI	0.52 ns	2.33 ns
Generation × Drought		
CZH	1.54 ns	1.81 ns
CZL	0.73 ns	3.08 **.**
FI	0.10 ns	0.39 ns

Chi‐square and significance from generalized linear mixed model tests.

All degrees of freedom = 1.

*p* values significance labels: ***p <0.001; .*p* <0.1; ns = not significant (*p* ≥0.1).

## 4 DISCUSSION

The aim of this study was first to evaluate the heritability of DNA methylation changes in response to environmental stimuli within apomictic dandelion lineages. In addition, we aimed at evaluating if the capacity for such inheritance is different in lineages that have successfully colonized medium‐altitude or high‐latitude habitats. In two apomictic dandelion lineages, drought stress showed marginally significant, accession‐specific direct stress effects on methylation profiles (accession × drought effect). But no transgenerational stability of induced DNA methylation changes was observed. No consistent pattern was observed that accessions from higher altitude or higher latitude sites are epigenetically more plastic than accessions from (presumably less stressful) low‐altitude or low‐latitude sites: in *T. hemicyclum*, drought stress showed a somewhat stronger DNA methylation response in the accessions that originate from higher latitude and altitude than in the lower latitude/altitude accession, but the opposite pattern was found in *T. alatum*. Salicylic acid, which mimics effects of defense induction by biotrophic pathogens, promoted seemingly undirected DNA methylation changes in offspring plants leading to an increase in DNA methylation variation (rather than a directed shift) in subsequent generations. This SA‐induced methylation increase in subsequent generations was not detectable in the stressed plants themselves, suggesting a more complex underlying mechanism of the plants’ response to SA than transgenerational stability of stress‐induced DNA methylation changes.

This study provides some support for the induction of DNA methylation modifications by environmental stresses, both as a direct effect in stressed plants and via an (unidentified) inherited effect causing novel changes in their unstressed progeny. Depending on genotype and environmental exposure, between 13% and 36% of the DNA methylation, changes observed in the first generation were stably inherited for at least two subsequent offspring generations, indicating the potential for epigenetic divergence within apomictic lineages. However, this estimate includes spontaneous DNA methylation changes that are unrelated to the environmental signal, and the effect of experimental treatments was generally weak, genotype‐dependent, environment‐specific and may involve different underlying mechanisms. An important observation from this study is that considerable levels of heritable DNA methylation variation build up irrespective of environments from generation to generation in this apomictic system.

Field studies in various plant species have revealed associations between methylation variation and biotic, as well as abiotic characteristics of the habitat (Foust et al., 2016; Gao, Geng, Li, Chen, & Yang, 2010; Gugger, Fitz‐Gibbon, PellEgrini, & Sork, 2016; Herrera & Bazaga, 2010; Lira‐Medeiros et al., 2010). Such associations may arise partly from environmental induction of DNA methylation variants, which can leave a functional “stress memory” in offspring of stressed plants (Wibowo et al., 2016). However, current evidence from *Arabidopsis thaliana* suggests that such functional environment‐directed DNA methylation variants often do not persist past the first offspring generation (Hagmann et al., 2015; Wibowo et al., 2016). For instance, in one widespread *A. thaliana* haplotype, Hagmann et al. (2015) observed that heritable DNA methylation differences accumulated rather in a stochastic manner, like genetic divergence, while environmentally induced effects were rarely inherited and did not contribute significantly to durable genomewide heritable epigenetic variation. Our results are consistent with these observations: We observed a clear buildup of DNA methylation variation over generations irrespective of stress treatments, and an additional inherited effect of stress exposure was expressed as an enhanced rate of DNA methylation changes per se (possibly stochastic) rather than as a clear directional shift in DNA methylation.

Our study used MS‐AFLPs to detect DNA methylation changes. This method can detect DNA methylation differences between samples but only few genomic loci are covered and the method provides no sequence‐based information that could shed light on their functionality. Therefore, if stress‐induced DNA methylation changes are restricted to few functional loci in the genome, then it is likely that such changes are missed. We recently observed in the same experimental samples that the G1 stress exposure leaves a footprint in the small RNA composition of the G3 generation, in a way that suggests transgenerational regulation of stress‐related genes (Morgado et al., 2017). Such a transgenerational signal in small RNAs might be mediated by stable inheritance of stress‐induced DNA methylation variants that affect small RNA production in subsequent generations—but such variants may have been missed by our MS‐AFLP screening approach. For future methylome screenings in experiments using large sample sizes that are typical for ecological population studies, we suggest to follow recently published methods using RADseq and Genotyping by Sequencing (Trucchi et al., 2016; Van Gurp et al., 2016). These methods make RRBS, reduced representation bisulfite sequencing (Meissner et al., 2005), cost‐effective for large sample sizes as well as for species without a priori knowledge of the genome.

One important factor in assessing the ecological and evolutionary relevance of epigenetic variation is to distinguish autonomous epigenetic variation from epigenetic variation that has a genetic basis. A recent study showed that even small genetic differences can be responsible for extensive genomewide DNA methylation differences (Dubin et al., 2015). In *Arabidopsis,* it has been shown that heat stress results in different phenotypic responses depending on the genotype as well as the tissue tested and the stress response was shown to persist for two generations only (Lang‐Mladek et al., 2010; Suter & Widmer, 2013). Such relations between genetic and epigenetic variation make it difficult to attribute adaptive potential to epigenetic variation alone. Strategies to address this problem include the use of statistical methods to distinguish patterns of epigenetic variation that are independent from patterns of genetic variation (Richards, Bossdorf, & Verhoeven, 2010) and the experimental use of completely inbred or asexually reproducing lineages (such as in this study). However, even in such inbred or clonal systems, when lacking high‐resolution genomic analysis, it is almost impossible to rule out underlying genetic variation as a factor. A genetic mechanism involved in epigenetic stress responses is for instance the regulation of transposable elements (TEs). TE transpositions, which are typically deleterious to the genome, are controlled by DNA methylations. The silenced state of TEs, which in turn can affect the expression of nearby genes, can persist through cell lines and across generations (Feng et al., 2010). Demethylations, and thereby the release of silenced TEs, have been shown in response to stress (Dowen et al., 2012; Grandbastien, 1998; Kalendar, Tanskanen, Immonen, Nevo, & Schulman, 2000; McCue, Nuthikattu, Reeder, & Slotkin, 2012), which can result in altered transcription of genes close to the TE and can generate genetic variation by the transposed TEs. The complex and ambiguous findings regarding the role and mechanism of epigenetic variation in plant populations call for more studies that link the causes and consequences of DNA methylation and try to disentangle sequence‐independent effects from sequence‐mediated effects.

In contrast to a previous study on effects of SA stress in apomictic dandelion (Verhoeven, Jansen, et al., 2010) and *Arabidopsis thaliana* (Dowen et al., 2012), we could not detect clear direct stress‐induced methylation changes in the SA‐exposed plants themselves. The observed lack of a detectable response in the SA‐exposed generation might derive from the low‐resolution technique of MS‐AFLPs, which detects only a small fraction of methylation changes. Alternatively, our results might suggest that different underlying mechanisms are causing the varying SA stress responses. Our study shows that novel epimutations arose in the second and third generation after SA application. The mechanism for such a “delayed” effect of SA stress is unknown, but might be associated with heritably altered TE activity that causes continued transpositions and associated methylation changes in subsequent generations. The differences between the SA stress responses observed by Verhoeven, Jansen, et al. (2010) and this study might also be related to the age of the apomictic lineage used. Whereas the current study is based on natural apomictic genotypes, the genotype used in the previous study (AS34) was a synthetic apomict derived experimentally by crossing a sexually reproducing mother (diploid) with pollen from an apomictic father (triploid) and therefore underwent very recent hybridization and polyploidization. Such genomic events are associated with DNA methylation reprograming and TE release which might affect responses to environmental stresses (Salmon, Ainouche, & Wendel, 2005; Verhoeven, Van Dijk, et al., 2010).

Quite independent from stress‐induced effects, we observed methylation variation that built up increasingly over the three tested generations indicating a considerable background rate of heritable epimutations. This provides evidence that DNA methylations can be stably transmitted and maintained for at least two generations. The stochastic epimutations in the offspring of unstressed plants presumably arise through spontaneous epimutations, as has been observed in other plants across generations (Becker et al., 2011; Schmitz et al., 2011; Van der Graaf et al., 2015). However, it cannot be excluded that these epimutations are caused by a volatile signal emitted from neighboring SA‐treated plants in the same growing chamber. It has been shown that stressed plants can affect neighboring plants even across a certain distance (Park, Kaimoyo, Kumar, Mosher, & Klessig, 2007). Future studies should take volatile effects into account by separation of the treatment groups or additionally analyzing the initial DNA methylation pattern instead of deriving a consensus. Using a methylome and genome screening in *A. thaliana*, Becker et al. (2011) found a high number of stochastic epimutations but also a frequent reversion of epimutations and a dependency on where and which type of DNA methylation (CG, CHG) was addressed. However, recent novel analyses in the same system have called the reported high reversal rates and lack of long‐term stability into question (Van der Graaf et al., 2015). Regardless of its origin, the observed significant buildup of methylation variation over generations could play a relevant role for selection and adaptive responses within an apomictic lineage, provided that it can be stably transmitted and depending on its phenotypic consequences (Schmitz et al., 2011). Stochastic epimutations could potentially also result in epigenetic divergence between sub‐lineages within apomictic lineages over microevolutionary time, consistent with the accession differences that we observed within single apomictic lineages.

## 5 CONCLUSION

This study reveals that stress exposure can have effects on DNA methylation patterns in unexposed offspring plants, but also that such effects are relatively weak, highly context‐dependent, and not expressed as consistent predictable changes at specific loci but rather as an increase in seemingly stochastic DNA methylation variation between plants. A clear observation was that spontaneous epimutations added to a buildup of DNA methylation variation across generations, irrespective of stress environments. Epimutations have been shown to occur at much higher rates than genetic mutations, generating variation that is potentially visible to natural selection. This could underlie the epigenetic variation and ultimately the within‐apomict differentiation that we observed between the different natural accessions tested. To what extent this epigenetic divergence is fully independent on genetic deviance has yet to be shown.

## AUTHOR CONTRIBUTIONS

VP and KJFV conceived and designed the study. WHvdP and AB contributed to study design and interpretation of results. JK organized the plant sampling and identification. VP and CO collected the data and analyzed the data. VP wrote the manuscript with input from all authors.

## DATA ACCESSIBILITY

Data available from the Dryad Digital Repository: https://datadryad.org//resource/doi:10.5061/dryad.tf536

## CONFLICT OF INTEREST

None declared.
