# Supplementary material for: Increased transgenerational epigenetic variation, but not predictable epigenetic variants, after environmental exposure in two apomictic dandelion lineages
Source: Ecol Evol. 2018 Feb 19;8(5):3047–59. doi: 10.1002/ece3.3871 (PMC5838036; doi:10.1002/ece3.3871)
Supplement: Supplementary file 1 [file ECE3-8-3047-s001.docx]

| **Figure S1** Principal Coordinate Analysis (PCoA) based on MS-AFLP profiles of drought stressed (red) and control plants (black) in the first, stressed generation. Data is plotted per accession (CZH : triangle; CZL : circle; FI : rectangle) and for two apomictic dandelion lineages ( *T. alatum* and *T. hemicyclum*): data points (empty symbols), centroids per treatment group (filled circles)*,* distance to centroid (grey lines) and 1 standard deviation ellipse. |
| --- |
| 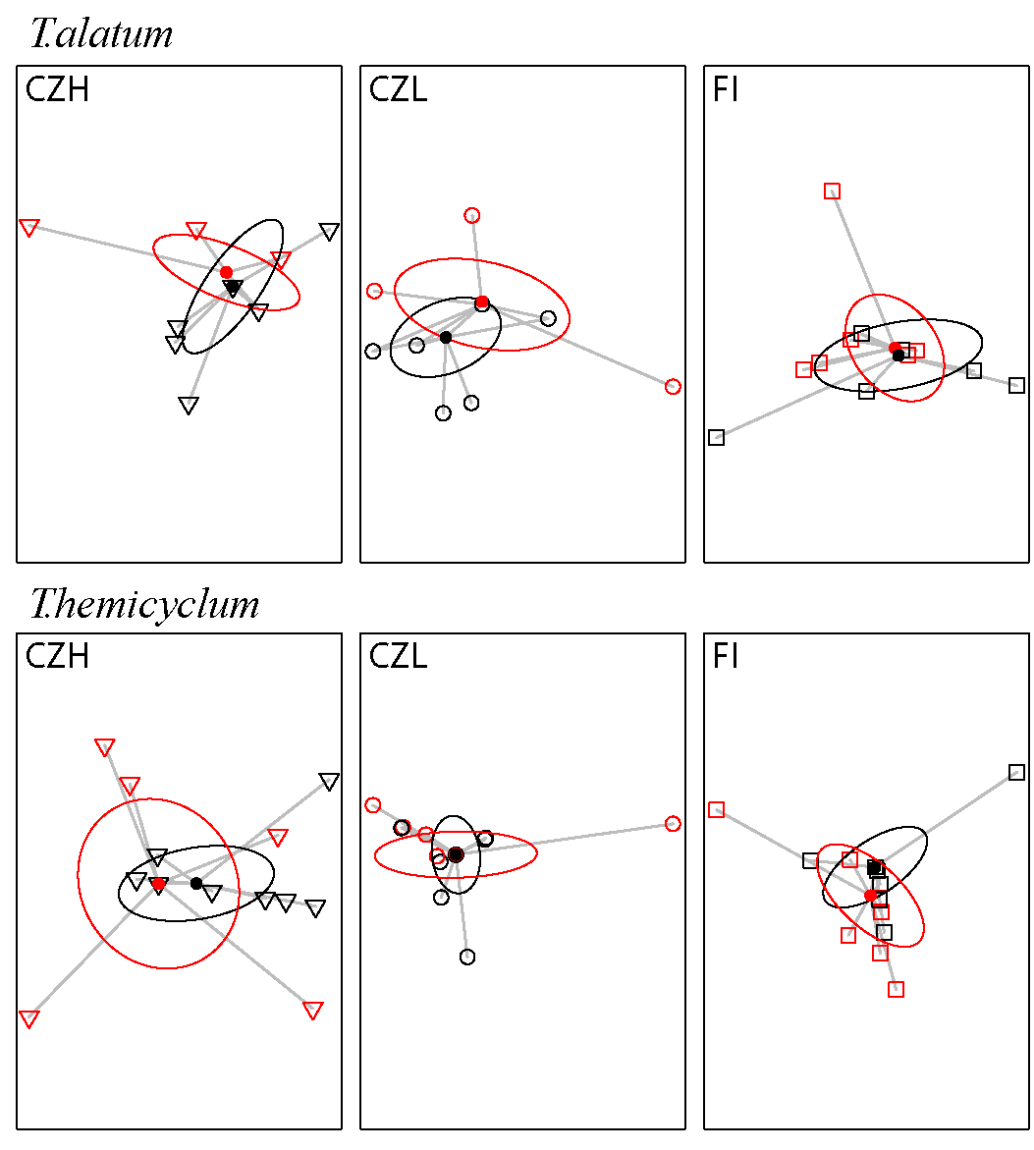 |

| **Figure S2** Principal coordinate Analysis (PCoA) based on MS-AFLP profiles of salicylic acid stressed (grey filled rectangles) and control plants (no fill) in the first, stressed generation and the unstressed progeny of the two subsequent generations. Data is plotted for two apomictic dandelion lineages (*T. alatum* and *T. hemicyclum)* from accession FI. |
| --- |
| **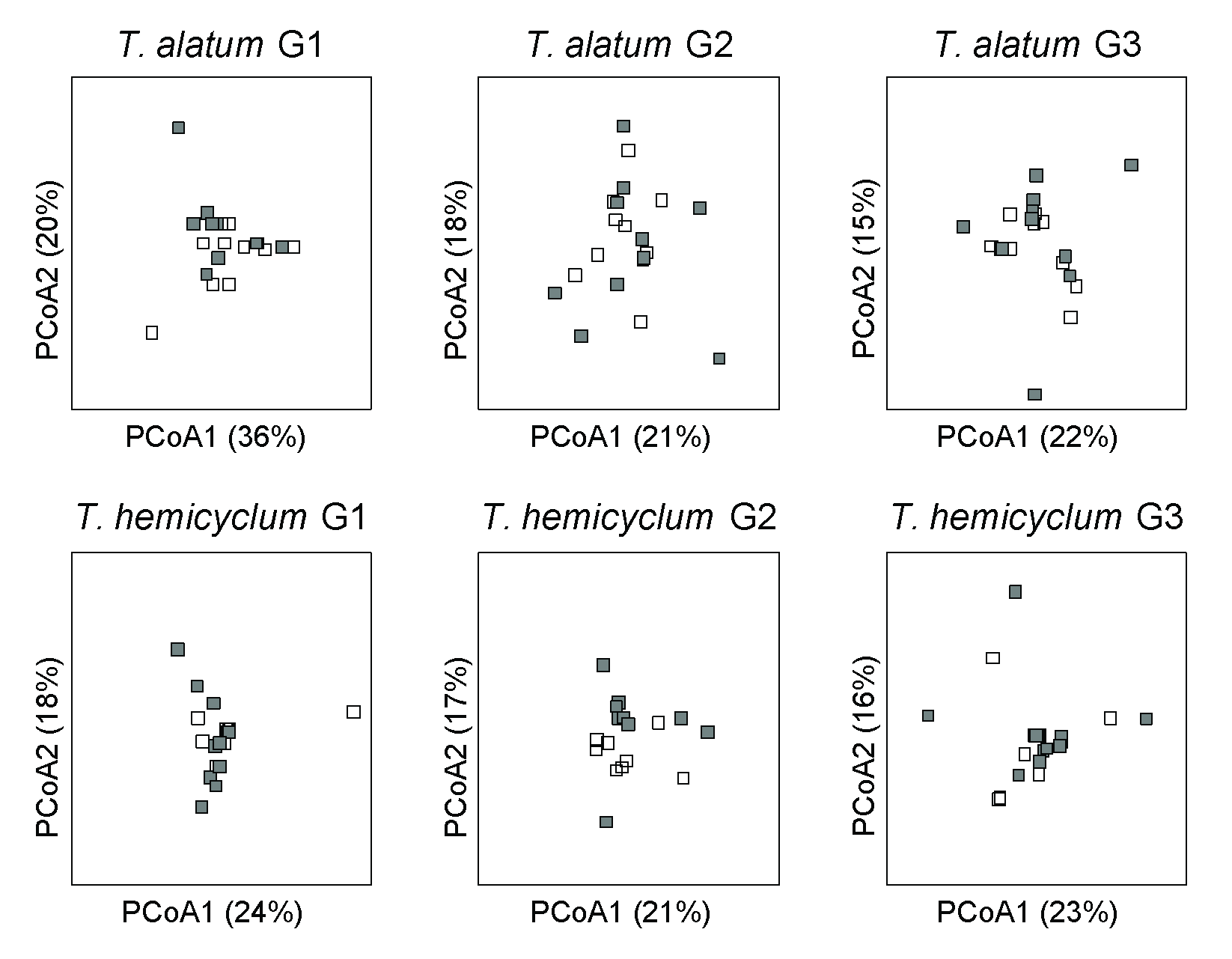** |

| **Table S1** Sampling sites of plant material | | | | | |
| --- | --- | --- | --- | --- | --- |
| Sampling site | Accession | Apomictic lineage | Latitude | Longitude | m.a.s.l. |
| Central Bohemia, CZ | CZL | *T. alatum* | 49° 50' 23'' N | 14° 31' 07'' E | 351 |
|  |  | *T. hemicyclum* | 50° 06' 31'' N | 15° 58' 14'' E | 302 |
| White Carpathians, CZ | CZH | *T. alatum* | 49° 16' 23'' N | 17° 58' 01'' E | 483 |
|  |  | *T. hemicyclum* | 49° 20' 13'' N | 18° 01' 17'' E | 373 |
| South East Finland | FI | *T. alatum* | 62° 11' 57'' N | 30° 35' 51'' E | 74 |
|  |  | *T. hemicyclum* | 62° 10' 29'' N | 30° 35' 47'' E | 77 |
|  | | | | | |

| **Table S2** Genotyping results from eight microsatellite markers per lineage (*T. alatum*, *T. hemicyclum*) and accession (CZL, CZH, FI) used for this experiment. | | | | | | | |
| --- | --- | --- | --- | --- | --- | --- | --- |
|  | *T. alatum* | | |  | *T. hemicyclum* | | |
|  | CZL | CZH | FI |  | CZL | CZH | FI |
| mst31 | 238 | 238 | 238 |  | 126 / 243 | 126 / 243 | 126 / 243 |
| mst44B | 185 | 185 | 185 |  | 176 / 195 | 176 / 195 | 176 / 195 |
| mst58 | 125 | 125 | 125 |  | 104 / 123 / 125 | 104 / 123 / 125 | 104 / 123 / 125 |
| mst61 | 136 / 138 | 136 / 138 | 136 / 138 |  | 131 / 134 / 145 | 131 / 134 / 145 | 131 / 134 / 145 |
| mst67 | 203 / 221 | 203 / 221 | 203 / 221 |  | 230 / 239 / 241 | 230 / 239 / 241 | 230 / 239 / 241 |
| mst72 | 175 / **201** | 175 / **211** | 175 / **209** |  | 176 / 186 / 192 | 176 / 186 / 192 | 176 / 186 / 192 |
| mst78 | 164 / 172 | 164 / 172 | 164 / 172 |  | 164 / 168 | 164 / 168 | 164 / 168 |
| mst143 | 238 / 240 / 246 | 238 / 240 / 246 | 238 / 240 / 246 |  | 238 / 246 | 238 / 246 | 238 / 246 |
| The sizes [bp] of the allele fragments per microsatellite marker show an almost uniform pattern per lineage (expectation in mst72 in *T. alatum*, bold numbers). | | | | | | | |

| **Table S3** Adapters and primers used for MS-AFLPs | | |
| --- | --- | --- |
| **Adapters^*^** |  | **Sequence 5´- 3´** |
| *Eco*RI-adapter I |  | CTCGTAGACTGCGTACC |
| *Eco*RI-adapter II |  | AATTGGTACGCAGTC |
| *Hpa*II Adapter I |  | GATCATGAGTCCTGCT |
| *Hpa*II Adapter II |  | CGAGCAGGACTCATGA |
| **Pre-selective primers** |  | **Sequence 5´- 3´** |
| *Eco*RI-A |  | GACTGCGTACCAATTC**A** |
| *Eco*RI-T |  | GACTGCGTACCAATTC**T** |
| *Hpa*II -T |  | ATCATGAGTCCTGCTCGG**T** |
| **Selective primers** |  | **Sequence 5´- 3´** |
| *Eco*RI + AAC/ACA/AG/ACC |  | GACTGCGTACCAATTC**AAC/ACA/AG/ACC** |
| *Hpa*II + TCA/TAC/TAG |  | ATCATGAGTCCTGCTCGG**TCA/TAC/TAG** |
| ^*^*Eco*RI adapters (Reyna-Lopez *et al.* 1997), *Hpa*II adapters (Xiong *et al.* 1999). Following eight *Eco*RI*/Hpa*II primer combinations were used: ACA / TAC, ACA / TCA, AAC / TAG, AG / TCA, AG / TAC, ACC / TCA, ACC / TAG, ACC / TAC | | |

**References**

Reyna-Lopez G, Simpson J, Ruiz-Herrera J (1997) Differences in DNA methylation patterns are detectable during the dimorphic transition of fungi by amplification of restriction polymorphisms. *Molecular and General Genetics*, **253**, 703-710.

Xiong L, Xu C, Maroof MS, Zhang Q (1999) Patterns of cytosine methylation in an elite rice hybrid and its parental lines, detected by a methylation-sensitive amplification polymorphism technique. *Molecular and General Genetics*, **261**, 439-446.
